# Bacterial feeding induces changes in immune-related gene expression and has trans-generational impacts in the cabbage looper (*Trichoplusia ni*)

**DOI:** 10.1186/1742-9994-6-7

**Published:** 2009-05-07

**Authors:** Dalial Freitak, David G Heckel, Heiko Vogel

**Affiliations:** 1 Max Planck Institute for Chemical Ecology, Department of Entomology, Hans Knöll Strasse 8, 07745 Jena, Germany

## Abstract

**Background:**

Poly- and oligophagous insects are able to feed on various host plants with a wide range of defense strategies. However, diverse food plants are also inhabited by microbiota differing in quality and quantity, posing a potential challenge for immune system mediated homeostasis in the herbivore. Recent studies highlight the complex interactions between environmentally encountered microorganisms and herbivorous insects, pointing to a potential adaptational alteration of the insects' physiology. We performed a differential gene expression analysis in whole larvae and eggs laid by parents grown on different diets to identify potential novel genes related to elevated microbial content in the caterpillars' food.

**Results:**

We used GeneFishing, a novel differential display method, to study the effects of dietary bacteria on the general gene expression in different life stages and tissues of the cabbage looper (*Trichoplusia ni*). We were able to visualize several hundred transcripts on agarose gels, one fifth of which were differentially expressed between treatments. The largest number of differentially expressed genes was found in defense-related processes (13) and in recognition and metabolism (16). 21 genes were picked out and further tested for differential gene expression by an independent method (qRT-PCR) in various tissues of larvae grown on bacterial and bacteria-free diet, and also in adults. We detected a number of genes indicative of an altered physiological status of the insect, depending on the diet, developmental stage and tissue.

**Conclusion:**

Changes in immune status are accompanied by specific changes in the transcript levels of genes connected to metabolism and homeostasis of the organism. Our findings show that larval feeding on bacteria-rich diet leads to substantial gene expression changes, potentially resulting in a reorganization of the insects' metabolism to maintain organismal homeostasis, not only in the larval but also in the adult stage. Furthermore, differences in gene expression levels can also be seen in the next generation, strongly influenced by parental diet.

## Background

Most Lepidopteran larvae are herbivorous and many among them are important pests in agriculture, causing severe damage to various crop plants growing in monocultures. The level of specialization even within a Lepidopteran family can vary dramatically. Larval feeding can be restricted to a specific plant part, like leaf material only or it can be extended to allow exploiting various plants including different parts of the plant (e.g. leaves, stem, flowers, and fruits) as a food source. In addition to the enormous variation in defensive proteins and secondary metabolite production, different parts of the plant are inhabited by different microorganisms [1]. Feeding on different plants and plant organs or even moving up and/or down on the leaves of the same plant is accompanied by potential changes in the ingested microflora, both qualitatively and quantitatively. Previously [2] we showed that feeding on large amounts of essentially non-pathogenic bacteria causes substantial changes in the immune status of larvae of the cabbage looper (*Trichoplusia ni*). Changes can be seen in immune response related enzyme activities and protein expression in the hemolymph, but also in transcription of immune-related genes in midgut tissue. Moreover, fitness related traits are impaired in animals due to ingestion of large amounts of bacteria in comparison to larvae feeding on sterile diet.

The mounting of immune responses is costly [3] and can result in severe autoimmune effects in insects [4,5]. However, very little is known about the accompanying changes in metabolic processes and the physiology of insects in the course of immune responses. This probably stems from the fact that researchers have mostly focused on known immune effectors and have also often restricted their analysis to direct immune repertoire cells, like hemocytes. A number of physiological changes taking place in the body during any immune insult may not be directly linked to the immune system, but to dealing with harmful side effects of the targeted immune response, allowing the organism to maintain homeostasis under stressful conditions.

An increasing amount of genomic data is accumulating for numerous invertebrates, as whole genome sequences are available now for honey bee (*Apis mellifera*) [6], fruitfly (*Drosophila melanogaster*) [7], mosquito (*Anopheles gambie*) [8], and the flour beetle (*Tribolium castaneum*) [9], and this has led to the flourishing of comparative immunology as an approach to study host-parasite interactions. Although the screening of various EST libraries and comparing strictly immune induced markers has revealed much information about immunity, this approach is based on previously identified genes from other organisms. This leads to the situation where it is hard to study new factors associated with a changed immune status, not necessarily directly involved in classical comprehension of the immune response. Furthermore, most studies focus on strictly pathogenic interactions. We therefore applied a random screening approach to identify novel genes involved in immune status changes of *T. ni*. We chose the GeneFishing method, a novel differential display technique, in order to study differential gene expression in a system with very little prior DNA sequence information.

In our study we examined global gene expression level differences, dependent on the dietary conditions of an herbivorous Lepidopteran larva. Transcripts of two and seven day old larvae grown on plants, on bacteria-supplemented and on non-supplemented artificial diet were compared. In addition we wanted to address the question whether parental diet can induce changes in gene expression in the following generation. To accomplish that, we compared the transcripts of eggs laid by parents grown on bacterial and bacteria-free diet. We show that changes in immune status are accompanied by specific changes in the transcript levels of genes connected to metabolism and homeostasis of the organism.

## Methods

### Animals

Cabbage semilooper (*Trichoplusia ni*) eggs were obtained from Entopath Inc. (USA). Larvae of *Trichoplusia ni *were grown on three different diets at room temperature (23°C) and a 16/8 h light/dark cycle, and at 55% relative humidity.

To estimate the impact of bacteria in the diet, three feeding groups were formed: larvae were fed on artificial diet [2] with or without bacteria (later referred to as bacterial and bacteria-free diet) and on cabbage plants (*Brassicae oleraceae*). Bacteria-free diet and plants may in fact contain low levels of environmental bacteria. Bacterial diet was specifically enriched for bacteria by soaking with overnight cultures (OD600 = 4) (2.5 ml/40 cm^2^) of *Escherichia coli *and *Micrococcus luteus *(~80 μg per 125 g of diet). Diets were changed every three days to keep the bacterial concentration in the diet at approximately the same level. To equalize the amount of handling stress between the plant diet and artificial diets, all the larvae feeding on cabbage plants were resettled on new plants also with the interval of three days.

To study the effect of parental diet on the gene expression in the eggs, two crosses were set up. Adults grown on bacteria-free diet were mated with each other (N♀N♂) and adults grown bacterial diet (B♀B♂) were crossed with each other. Matings were carried out in 21 × 13 × 13 cm cages. Three day old eggs were collected and used for further expression analyses.

### Differential gene expression analysis

To study differential gene expression between *T. ni *larvae grown on plant, bacterial and bacteria-free diet the DEG GeneFishing Kit (SeeGene, Seoul) was used, following the manufacturer's protocol. Briefly, 3 μg of DNA-free total RNA was converted into single-stranded DNA using annealing control primer one (dTACP1) and two (dTACP2) which prime from the polyA tail, and a mixture of different reverse transcriptases (Array Script, Ambion; Bioscript, Bioline). Second-strand cDNA synthesis and subsequent PCRs were performed essentially as described in the DEG GeneFishing protocol. We used a total of 40 different ACP (annealing control primer) pairs. ACP primers are designed for highly specific PCR in a two-stage process. In the first stage, amplification is based on a perfect match between the short 3' end of the ACP and the cDNA template; and the second more stringent stage further amplifies the specific product based on pairing of the longer 5' end of the ACP to a additional primer; with no further nonspecific amplification from the cDNA template. Like all differential display techniques, this method identifies a fraction of the total gene expression changes, depending on the number of different primers employed.

**Figure 1:**
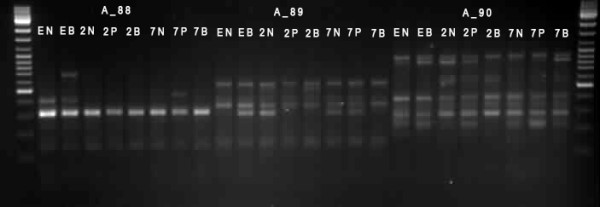
**Example of gel with separated PCR products from differential gene expression analysis**. (2B – 2 day old larvae on bacterial diet, 2N – 2 day old larvae on bacteria-free diet, 2P – 2 day old larvae on cabbage plants; 7B – 7 day old larvae on bacterial diet, 7N – 7 day old larvae on bacteria-free diet, 7P – 7 day old larvae on cabbage plants; EN – eggs laid by parents grown on bacteria-free diet, EB – eggs laid by parents grown on bacterial diet). A_88, A_89, A_90 are primer pairs used for GeneFishing analysis.

PCR products were separated and visualized on 2% agarose gels. Bands were scored visually and differentially expressed bands were cut out from the agarose gels and PCR products were extracted using the Zymoclean Gel DNA Recovery Kit ™ (Zymo Research) according to the manufacturer's instructions (Figure 1). DNA fragments were cloned into the TOPO TA cloning vector (Invitrogen) following the manufacturer's protocol. For identification of inserted DNA, isolated plasmids were sequenced (Applied Biosystems, ABI). BLAST searches were conducted on a local server using the National Center for Biotechnology Information (NCBI) Blastall program and best hits were recorded.

To assess the amount of internal variation within treatments, we selected two treatments, formed three different pooled samples for each, and used 20 ACP primer pairs to search for variation among the samples from the same treatment group. No differences were found for this subset (data not shown); therefore we used a single superpool for each treatment group in subsequent analysis.

### RNA isolation

Dissected insect midguts and the rest of the bodies (except for the head capsules) were ground using a motorized hand pestle and total RNA was isolated using the TRIzol Reagent (Invitrogen) according to the manufacturers' protocol. To isolate RNA from whole larvae, eggs and adults, tissues were submersed in liquid nitrogen and homogenized using mortar and pestle, the powder was dissolved in TRIzol Reagent (Invitrogen) and RNA was extracted according to the standard protocol. An additional DNAse (Turbo DNAse, Ambion) treatment was included prior to the second purification step to eliminate any contaminating DNA. A second purification step was performed with RNeasy MinElute CleanUp Kit (Qiagen). RNA integrity was verified on an Agilent 2100 Bioanalyzer using RNA Nano chips (Agilent). RNA quantity was determined photospectrometrically using a NanoDrop ND-1000 Spectrophotometer (Thermo Scientific).

### Quantitative real-time PCR

500 ng of DNA-free total RNA was converted into single-stranded cDNA using a mix of random and oligo-dT20 primers according to the ABgene protocol (ABgene). Real-time PCR oligonucleotide primers were designed using the online Primer3 internet based interface . Primers were designed by the rules of highest maximum efficiency and sensitivity rules were followed to avoid formation of self and hetero-dimers, hairpins and self-complementarity. Gene-specific primers were designed on the basis of sequences obtained for selected *T. ni *genes and several additional genes as potential house-keeping genes to serve as the endogenous control (normalizer) [see additional file 1]. Q-RT-PCR was done in optical 96-well plates on a MX3000P Real-Time PCR Detection System (Stratagene) using the Absolute QPCR SYBR green Mix (ABgene) to monitor double-stranded DNA synthesis in combination with ROX as a passive reference dye included in the PCR master mix.

## Results and discussion

### Differential gene expression analysis in dietary challenged *T. ni *larvae and eggs

We examined gene expression patterns using GeneFishing, a PCR-based differential display technique. Analysis was carried out using purified RNA from 2 and 7 day old whole larvae and eggs. After 40 PCR cycles, agarose gel electrophoresis revealed cDNA bands ranging in size from 100 bp to 2.5 kb. Using a combination of 60 arbitrary ACP primers and two anchored oligo(dT) primers, a total of 323 bands were visualized on agarose gels. 60 bands were identified as differentially expressed between larvae fed on three different diets and those bands were cut out of the agarose gels, purified, cloned and sequenced. In the case of eggs, 31 differentially expressed bands were identified out of 141 total bands. Obtained sequences were compared to the National Center for Biotechnology Information databases using a blastx program (Table 1).

**Table 1:** cDNAs from GeneFishing for *Trichoplusia ni*.

**Cluster**	**Acc. No**	**Best BlastX match**	**e value**
***Defense and recognition***	GH270329	ABQ43785: mitochondrial cyctochrome c oxidase subunit VIa [Bombyx mori]	3.00E-06
	GH270411	ABV68856.1: gloverin [Trichoplusia ni]	2.00E-07
	GH270330	NP_001106738: ubiquinol cytochrome c reductase [Bombyx mori]	2.00E-99
	GH270331	NP_001037299: Mn superoxide dismutase [Bombyx mori]	9.00E-95
	GH270389	NP_001040131.1: GST omega1 [Bombyx mori]	5.00E-52
	GH270405	ABV68857.1: Hdd1-like protein [Trichoplusia ni]	7.00E-14
	GH270332	P_001040251: lectin 4 C-type lectin [Bombyx mori]	1.00E-96
	GH270378	ACC91897.1: hemolin [Heliothis virescens]	5.00E-15
	GH270333	AQ75437: cathepsin L-like protease [Helicoverpa armigera]	7.00E-75
	GH270348	XP_974384: PREDICTED: similar to homologue of Sarcophaga 26, 29 kDa proteinase [Tribolium castaneum]	1.00E-33
	GH270334	NP_001106742: cytochrome c oxidase polypeptide Vb [Bombyx mori]	9.00E-47
	GH270337	AAS79891: gst1 [Spodoptera litura]	9.00E-11
	GH270339	P_001037057: BCP inhibitor [Bombyx mori]	2.00E-04

***Development***	GH270340	BAG30780: muscle protein 20 like protein [Papilio xuthus]	3.00E-09
	GH270341	XP_001662353: hypothetical protein AaeL_AAEL012245 [Aedes aegypti], conatins chitin binding domain	4.00E-39
	GH270382	XP_001988215: GH10690 [Drosophila grimshawi], contains chitin binding domain, Perithrophin A	5.00E-11
	GH270345	NP_001108405: titin1 [Bombyx mori]	4.00E-05

***Digestion***	GH270346	AAA29341: trypsin [Manduca sexta]	3.00E-40
	GH270365	BAD22559: lipase [Antheraea yamamai]	7.00E-50
	GH270347	AAV91434: serine protease 3 [Lonomia obliqua]	2.00E-52
	GH270394	P35045: Trypsin, alkaline A precursor	2.00E-39
	GH270343	XP_972363.2: similar to trypsin-like serine protease [Tribolium castaneum]	6.00E-122
	GH270376	XP_001650916: tyrosine-protein kinase [Aedes aegypti]	2.00E-33

***DNA related***	GH270368	NP_001040495: C14orf124 protein [Bombyx mori]	5.00E-20
	GH270385	XP_001952287: PREDICTED: similar to chromosome 19 open reading frame 29 [Acyrthosiphon pisum]	6.00E-22
	GH270400	XP_001200852: PREDICTED: similar to histone H2B (aa 1–123) [Strongylocentrotus purpuratus]	6.00E-07

***Metabolism***	GH270353	NP_001091831: enolase [Bombyx mori]	9.00E-58
	GH270417	XP_001960088: GF13192 [Drosophila ananassae], similar to short-chain dehydrogenase	4.00E-23
	GH270358	XP_552371: AGAP011872-PA [Anopheles gambiae str. PEST], ubiquitin activating enzyme	2.00E-66
	GH270375	EEA92944.1: alcohol dehydrogenase (acceptor) [Pseudovibrio sp. JE062]	4.00E-11
	GH270397	NP_001040294: protease inhibitor 1 [Bombyx mori]	8.00E-18
	GH270384	NP_001037171: protein disulfide isomerase [Bombyx mori]	2.00E-100
	GH270355	XP_396707: PREDICTED: similar to Serine/threonine-protein kinase polo [Apis mellifera]	4.00E-47
	GH270409	NP_001040233: ATP synthase [Bombyx mori]	7.00E-146
	GH270415	XP_001599579: PREDICTED: similar to ENSANGP00000017562 [Nasonia vitripennis], similar to stearoyl-coa desaturase	8.00E-28
	GH270418	NP_001040310: light-induced protein-like brain protein 44 [Bombyx mori]	2.00E-34
	GH270388	NP_001040436: hydroxysteroid dehydrogenase [Bombyx mori]	8.00E-56
	GH270407	XP_564957: Elongase AGAP007264-PA [Anopheles gambiae str. PEST]	2.00E-45
	GH270433	ABG77272: ubiquitin-53aa extension protein [Pieris rapae]	7.00E-05
	GH270423	NP_001037047: silk proteinase inhibitor [Bombyx mori]	4.00E-10
	GH270386	AAL60239: takeout [Aedes aegypti]	1.00E-08

***Ribosomal protein***	GH270406	ABS57435: ribosomal protein S25 [Heliconius melpomene]	1.00E-27
	GH270431	AAK92157: ribosomal protein L14 [Spodoptera frugiperda]	4.00E-06
	GH270381	AAN86048: ribosomal protein S2 [Spodoptera frugiperda]	4.00E-49
	GH270374	AAL26578: ribosomal protein S3 [Spodoptera frugiperda]	1.00E-51
	GH270396	AAV91399: ribosomal protein 27 [Lonomia obliqua]	4.00E-11
	GH270410	NP_001091753: ribosomal protein L36A [Bombyx mori]	2.00E-29
	GH270377	NP_001037570: ribosomal protein S15A [Bombyx mori]	2.00E-30
	GH270416	AAV91403: ribosomal protein 5 [Lonomia obliqua]	3.00E-26

***Signaling***	GH270352	NP_724186: Paxillin [Drosophila melanogaster]	1.00E-111
	GH270363	XP_974400: PREDICTED: similar to lots wife CG33968-PA, Nose resistant to flouoxetine family member [Tribolium castaneum]	8.00E-12
	GH270422	XP_001599614.1: signal peptidase 12kda [Nasonia vitripennis]	4.00E-07
	GH270420	NP_001040123: electron-transfer-flavoprotein beta polypeptide [Bombyx mori]	4.00E-32

***Unknown***	GH270366	XP_001604274.1: similar to CG12009-PA [Nasonia vitripennis]	3.00E-04
	GH270399	XP_001121582: PREDICTED: similar to CG10710-PA [Apis mellifera]	1.00E-06

***No significant match***	GH270336	YP_001329156: glyoxalase/bleomycin resistance protein/dioxygenase [Sinorhizobium medicae WSM419]	e>-1
	GH270350	XP_001421381: predicted protein [Ostreococcus lucimarinus CCE9901]	e>-1
	GH270359	XP_001759276: predicted protein [Physcomitrella patens subsp. patens]	e>-1
	GH270390	XP_001717683: PREDICTED: hypothetical protein [Homo sapiens]	e>-1
	GH270429	NP_001040436: hydroxysteroid dehydrogenase [Bombyx mori]	e>-1
	GH270401	P_001943585: PREDICTED: similar to zinc finger protein 624 [Acyrthosiphon pisum]	e>-1
	GH270403	EAW95139.1: serine arginine repetitive matrix 1 [Homo sapiens]	e>-1
	GH270428	YP_001744939: hypothetical protein EcSMS35_2915 [Escherichia coli SMS-3-5]	e>-1
	GH270387	NP_927355: hypothetical protein glr4409 [Gloeobacter violaceus PCC 7421]	e>-1
	GH270403	ZP_01465929: hypothetical protein STIAU_3143 [Stigmatella aurantiaca DW4/3-1]	e>-1
	GH270351	BAB89324: putative G-protein coupled receptor [Homo sapiens]	e>-1
	GH270356	XP_001647881: Gustatory receptor 61a, putative [Aedes aegypti]	e>-1
	GH270389	ABU41034: hypothetical protein [Lepeophtheirus salmonis]	e>-1
	GH270395	AAA26387: outer membrane protein A [Rickettsia akari]	
	GH270424	ZP_02747435: two-component sensor histidine kinase [Clostridium difficile QCD-63q42]	e>-1
	GH270342	XP_001867382: myosin I [Culex pipiens quinquefasciatus]	e>-1
	GH270362	YP_001910237: putative secretion/efflux abc transporter, ATP-binding protein [Helicobacter pylori Shi470]	e>-1
	GH270338	CAA43583: hydroxyproline-rich glycoprotein [Oryza sativa (indica cultivar-group)]	e>-1
	GH270335	XP_001952211: PREDICTED: similar to corneal wound healing-related protein [Acyrthosiphon pisum]	e>-1
	GH270344	XP_001017307: Chitinase class I family protein [Tetrahymena thermophila SB210]	e>-1
	GH270413	NP_508266: Serpentine Receptor, class H family member (srh-19) [Caenorhabditis elegans]	e>-1
	GH270391	XP_318947: Porin AGAP009833-PA [Anopheles gambiae str. PEST]	e>-1
	GH270404	XP_821286: hypothetical protein Tc00.1047053506401.350 [Trypanosoma cruzi strain CL Brener]	e>-1
	GH270367	YP_812104: hypothetical protein LACR_2573 [Lactococcus lactis subsp. Cremoris SK11]	e>-1
	GH270426	XP_001652430: hypothetical protein AaeL_AAEL001147 [Aedes aegypti]	e>-1
	GH270432	ZP_01222271: hypothetical protein P3TCK_18689 [Photobacterium profundum 3TCK]	e>-1
	GH270357	XP_976032: PREDICTED: hypothetical protein [Tribolium castaneum]	e>-1

***Hit only against EST***	GH270392	EB827756: 1151973 KZ03 Plodia interpunctella cDNA clone 584001, mRNA sequence	9.00E-08
	GH270360	Sf1F01923-3-1 Spodoptera frugiperda Fat Body cDNA Library Spodoptera frugiperda cDNA, mRNA sequence	4.00E-12
	GH270373	CF258147: 90 Trichoplusia ni fifth instar digestive system cDNA library Trichoplusia ni cDNA, mRNA sequence	6.00E-63
	GH270371	EY265223: BF01036X1D07.f1 Normalized subtracted keck library BF01 Danaus plexippus cDNA clone BF01036X1D07.f1 5, mRNA sequence	8.00E-07
	GH270425	AT001017: AT001017 Bombyx mandarina library (Hwang JS) Bombyx mandarina cDNA clone H340, mRNA sequence	2.00E-19
	GH270354	TN-LN-384-G-03-libF_J07 Trichoplusia ni whole larvae normalized cDNA library	7.00E-85
	GH270398	FF375565: TN-28-lipF_D07 Trichoplusia ni larval non-normalized cDNA library Trichoplusia ni cDNA clone TN-28-lipF_D07 5', mRNA sequence	6.00E-42
	GH270412	FF377777: TN-58-pDNR-lipF_N24 Trichoplusia ni larval non-normalized cDNA library	5.00E-16
	GH270419	FF377954:TN-60-pDNR-lipF_N13 Trichoplusia ni larval non-normalized cDNA library Trichoplusia ni cDNA clone TN-60-pDNR-lipF_N13 5', mRNA sequence	6.00E-72
	GH270379	EX212089: EST # 0000117 Spodoptera litura oxidative-stress responsive cDNA library Spodoptera litura cDNA clone SL-470, mRNA sequence	2.00E-50

***No hit at all***	GH270393	No hit against EST databases as well	
	GH270408	No hit against EST databases as well	
	GH270372	No hit against EST databases as well	
	GH270427	No hit against EST databases as well	
	GH270364	No hit against EST databases as well	
	GH270421	No hit against EST databases as well	
	GH270349	No hit against EST databases as well	
	GH270369	No hit against EST databases as well	
	GH270370	No hit against EST databases as well	
	GH270414	No hit against EST databases as well	

Shown are the accession numbers and the best BLASTX hits with e-values for each gene. Results are divided into different clusters, based on their physiological function.

Like all differential display techniques, the GeneFishing method can be expected to reveal a subset of expression differences; and may be biased towards more abundant transcripts. Although relatively new, it has already been applied to studies of animal neurophysiology [10], cancer [11] and plant allellopathy [12] and development [13]. So far a quantitative evaluation of the technique compared with others such as cDNA-AFLP, RAPD-differential display, or microarray analysis has not been carried out. Thus we selected 21 genes for further testing by an independent method (qRT-PCR) for differential gene expression in various tissues of larvae grown on bacterial and bacteria-free diet, and also in adults.

### Physiological changes upon feeding on different diets in larvae

We were able to identify 102 differentially expressed genes among larvae grown on different diets (Table 1). Several of these genes show age dependent expression levels, being influenced by diet only at certain developmental stages. We divided the identified genes into eight functional categories/clusters – defense and recognition, development, digestion, DNA-related, metabolism, ribosomal proteins, signaling and genes with unknown function. In addition we also listed the transcripts which gave no significant hit to any known protein or expressed sequence tag (EST) library (Table 1). In total we were unable to identify 49 of our transcripts via Blast searches. The reasons we failed to identify a number of transcripts could be partially embedded in the approach we took for studying global gene expression patterns. Due to the methodology of the GeneFishing technique, we mainly amplify regions of the mRNA close to the polyA tail and the 3' UTR region of the transcript, which is not the most informative for identification of the gene, as it contains non-coding sequence. This method in combination with the lack of the sequence information when studying a non-model organism can lead to difficulties with gene identification.

When examining the transcripts involved in defense and recognition we found several strictly immune response related genes, but also general stress and detoxification related indicators (Table 1). The immunity related genes show a very interesting expression pattern when comparing larvae feeding on different diets. The immune inducible effector molecule gloverin [14] is highly abundant in 2 and 7 day old larvae fed on plant and bacterial diet [see additional file 2]. The expression patterns for hemolin and HDD1, both known to be part of the immune response and up regulated upon immune challenge and bacterial feeding [2], are more complex. We see higher expression of hemolin in 2 day old bacteria-free diet fed larvae and 7 day old bacterial and plant diet larvae. HDD1 is expressed in all the larval stages and eggs at the same level, with the exception of a higher expression in 7 day old bacterial diet fed larvae. Hemolin is an immune protein in Lepidoptera, participating in phenoloxidase mediated immune responses [15,16], and its silencing in *Hyalophora cecropia *pupae is lethal for the next generation [17]. Hemolin is supposedly also involved in antiviral defense [18], whereas the function of HDD1 in immunity is not really known so far [19]. A cathepsin L-like protease was up regulated in 2 day old larvae grown on plant and bacteria-free diet in comparison to bacterial diet fed larvae, and it was highly expressed in 7 day old bacteria fed larvae [see additional file 2]. Cathepsin L-like proteases are cystein proteases, which are known to participate in tissue remodeling during insect metamorphosis [20]. In the case of vertebrates, this enzyme is also known to be involved in immunological processes, and in the leech *Theromyzon tessulatum *cathepsin L is involved in direct immune responses [21]. We observed the down regulation of a C-type lectin receptor gene in 2 day old larvae fed on plants and in 7 days old larvae fed on bacterial diet [see additional file 2]. These lectin receptors are involved in antifungal immunity mediating fungal binding, uptake and killing, and are probably also contributing to initiation and/or modulation of the immune response of the whole organism [22]. Most interestingly we also observed the down regulation of C-type lectins, possibly implying that also general immune response related genes are influenced upon bacteria-rich diet. In case that lectins are also involved in antifungal defenses of *T. ni*, then down regulation of this type of defense-related genes could be caused by trade-offs between various immune system components, e.g. antibacterial vs. antifungal defenses. This is in good correlation with our previous findings, where we see several immune inducible genes up regulated in midgut tissue upon larval exposure to bacterial diet [2]. Two different cytochrome c related genes were up regulated in 2 and 7 days old larvae fed on plant and bacterial diet [see additional file 2]. Cytochromes c (cytC) are electron-transfer proteins, having one or more heme c groups attached to proteins. Cytochromes c possess a wide range of properties and function in a large number of different redox processes, present in bacteria and mitochondria [23,24]. Seven day old plant feeding larvae also show a higher expression of a glutathione S-transferase (GST) when compared to the other diets. GST proteins are known to be involved in insecticide and plant toxin detoxification. The up regulation of a GST in the larvae feeding on plants could thus be explained by a strict correlation with the detoxification of plant secondary compounds [25] rather than a response to the bacterial load of the plant material. We can assume that our observation of the up regulation of several defense reaction-related transcripts in the larvae feeding on plant and bacterially challenged diet to be symptomatic of increased stress levels for the organism. Up regulation of immune responses is considered costly, as it often involves the release of multiple, potentially cytotoxic molecules, which in turn can cause activation of other stress related defenses [26-28]. This crosstalk between different biotic and abiotic stressors can lead to a significant overlap of the resulting complex transcriptional changes.

Among the genes related to insect development we identified a member of the chitinase gene family and two genes involved in muscle development. There is no clear diet dependent expression pattern of these genes [see additional file 2]. Differences in the expression of genes involved in developmental processes are possibly related to the physiological costs for feeding on nutritionally (plant *vs*. artificial diet) and microbially (bacterial *vs*. bacteria-free diet) different diets. These might be associated to delayed development and reduced pupal masses, as could be seen in our previous study [2]. Such negative effects in life-history traits are often accompanied with reduced reproductive success [29].

The differential gene expression analysis revealed altered expression levels of various digestive protein genes depending on the diet (Table 1). Major differences could be observed when comparing larvae fed on plant diet and both artificial diets. Both a trypsin and a lipase show a higher expression level in 7 day old larvae fed on plant diet and trypsin also in the 2 day old larvae fed on similar diet [see additional file 2]. These changes in expression levels could be linked to the occurrence of protease inhibitors in leaf tissue [30] or to overall differences in lipid and protein concentrations in the plant tissue in comparison to the optimal artificial diet. There is no clear presence or absence pattern in the expression of genes coding for digestive enzymes, but differences in expression levels. Bacterial diet and bacteria-free diet show no differences in the expression of digestive enzyme coding genes, with the exception of one trypsin, which is expressed only in bacteria fed larvae. Thus the costs of fitness-related traits, seen with larvae feeding on bacterial and bacteria-free larvae [2] are probably not directly linked to a complete remodeling of the digestive processes.

Several metabolism related genes were identified as differentially expressed in our analysis. Enolase was found to be highly expressed in 2 and 7 day old larvae fed on bacterial diet. Enolase is a metalloenzyme with catalytic activity involved in glycolysis and is present in all tissues and organisms capable of glycolysis or fermentation [31]. 2 day old larvae fed on bacterial diet had higher mRNA levels for a glucose-methanol-choline oxidoreductase (GMC oxidoreductase) and protease inhibitor 1. GMC oxidoreductases belong to a large family of diverse FAD enzymes. In insects these enzymes are often involved in the regulation of common developmental and physiological processes related to ecdysteroid metabolism [32] but in many cases their functions are still unknown. Protease inhibitors are a class of proteins involved in regulating the activity of various endogenous and exogenous proteases. However, they can also have a role in digestive processes, as well as defense or development [30]. We could also identify a number of enzymes involved in ATP binding and synthesis and several ribosomal proteins to be differentially expressed in 2 and 7 day old larvae grown on different diets [see additional file 2]. When examining the expression pattern of genes putatively identified as proteins involved in signaling, we detected a general trend of higher expression levels in plant and bacterial diet fed larvae [see additional file 2]. The changes in genes involved in general metabolism advocate serious constraints exerted on the organism by alteration of the quality of the diet plant vs. artificial but also bacterial vs. bacteria-free diet.

### Tissue-specific differential gene expression

To examine the expression of a selected subset of genes more closely within the organism, we used midguts and non-midgut tissue (the rest of the body) of 9 day old *T. ni *larvae grown on bacterial and bacteria-free diet. We found a number of genes to be down regulated in the midgut tissue of bacteria fed larvae, namely GMC oxidoreductase, lectin 4, a protease inhibitor and titin1. Glutathione S-transferase 1 was the only gene we found to be down regulated in the rest of the body of bacteria-fed larvae, whereas GMC oxidoreductase, a putative G-protein coupled receptor, alcohol dehydrogenase, tyrosine-protein kinase and a serine protease were up-regulated (Table 2). The alcohol dehydrogenase belongs to the short-chain dehydrogenases/reductases (SDR), a large enzyme family, most of which are known to be NAD- or NADP-dependent oxidoreductases [33]. These alterations are possibly connected with an elevated immune status [2] caused by feeding on bacteria rich diet and could be directed to deal with harmful side effects of the elevated immune status and/or help in the case of possible infection.

**Table 2:** RT-qPCR results for 9 day old larval midguts and non-midgut tissue (restbody) for genes identified by GeneFishing.

**Gene**	**Midgut**	**ct value (Bac)**	**Restbody**	**ct value (Bac)**
***Defense***				
cathepsin L-like protease	1.77 ± 0.018	19.99	1.17 ± 0.034	19.22
cytochrome c oxidase polypeptide Vb	1.07 ± 0.074	17.81	-1.08 ± 0.094	18.61
mitochondrial cytochrome c oxidase subunit VIa	-1.02 ± 0.115	17.93	-1.15 ± 0.004	19.3
Mn superoxide dismutase	-1.03 ± 0.026	20.18	-1.75 ± 0.030	20.38
lectin 4 C-type lectin	**-3.49 ± 0.048**	25.53	-1.96 ± 0.129	21.9
Glutathion S-transferase 1	1.80 ± 0.037	20.35	**-9.17 ± 0.058**	27.94
ubiquinol-cytochrome c reductase	-1.16 ± 0.005	19.27	-1.10 ± 0.014	19.74

***Development***				
trypsin-like serine protease	-1.35 ± 0.212	32.96	**2.02 ± 0.037**	23.89
hypothetical protein AaeL_AAEL012245, contains chitin binding domain	-1.29 ± 0.210	31.98	-1.42 ± 0.163	31.89
Perithrophin A	**-78.25 ± 0.067**	29.15	-1.28 ± 0.148	28.17
myosin I	-1.27 ± 0.014	22.73	-1.72 ± 0.035	22.47
titin1	**-2.32 ± 0.018**	20.1	1.29 ± 0.028	16.64

***Digestion***				
tyrosine-protein kinase	-1.40 ± 0.134	25	**3.96 ± 0.007**	28.89

***DNA related***				
C14orf124 protein	-1.03 ± 0.023	22.48	-1.15 ± 0.030	21.59
zinc finger protein	-1.05 ± 0.065	21.13	-1.08 ± 0.267	21.97

***Metabolism***				
alcohol dehydrogenase (acceptor)	**-2.05 ± 0.939**	19.8	**2.25 ± 0.062**	22.76
protease inhibitor 1	**-2.45 ± 0.325**	29.81	-1.18 ± 0.131	19.04
Serine/threonine-protein kinase polo	-1.45 ± 0.097	31.06	-1.17 ± 0.048	26.2
short-chain dehydrogenase	-1.20 ± 0.018	21.09	**6.12 ± 0.117**	26.29
protein disulfide isomerase	-1.09 ± 0.078	17.63	1.25 ± 0.074	17.66

***Signaling***				
putative G-protein coupled receptor	-1.40 ± 0.25	19.31	**9.48 ± 0.129**	25.49

Results are shown for animals grown on bacterial diet, where bacteria-free diet treatments were set to 1 (values are mean ± SD).

### Differential gene expression in *T. ni *adults

21 selected genes were also examined for differential expression in pooled adult female and male insects, and three were found to differ in expression levels between the diet the larvae had encountered. A putative chitin binding protein was down regulated and an alcohol dehydrogenase up regulated (Table 3) if pooled mRNA from both sexes was examined. In addition, we studied the expression of some of the genes in both sexes separately, and we could find higher levels of cytochrome c oxidase and alcohol dehydrogenase transcripts in bacteria-fed males. With the exception of these three genes we were not able to see any differential expression in genes selected from our GeneFishing analysis in the adult stage of *T. ni*. The relatively small number of differentially expressed genes found in the adult stage could be due to the fact that we have pre-selected gene candidates based on expression data in larvae, and the physiological requirements for adult moths are quite different from those of larvae.

**Table 3:** RT-qPCR results for adults fed on different diets for genes identified by GeneFishing.

**Gene**	**Genders pooled**	**Females**	**ct value**	**Males**	**ct value**
***Defense***					
cathepsin L-like protease	1.01 ± 0.100	1.02 ± 0.046	18.23	**2.16 ± 0.158**	17.46
cytochrome c oxidase polypeptide Vb	1.41 ± 0.010				
mitochondrial cytochrome c oxidase subunit VIa	1.29 ± 0.045				
Mn superoxide dismutase	1.27 ± 0.050	-1.01 ± 0.036	19.87	1.89 ± 0.097	19.57
lectin 4 C-type lectin	1.47 ± 0.005				
Glutathion S-transferase 1	1.04 ± 0.310	1.59 ± 0.258	30.63	1.54 ± 0.236	30.38
ubiquinol-cytochrome c reductase	1.01 ± 0.070				

***Development***					
trypsin-like serine protease	-1.19 ± 0.040				
hypothetical protein AaeL_AAEL012245, contains chitin binding domain	**-2.91 ± 0.240**	-1.43 ± 0.050	27.8	1.55 ± 0.015	28.75
Perithrophin A	-1.08 ± 0.115				
myosin I	-1.18 ± 0.115				
titin1	-1.42 ± 0.090	-1.06 ± 0.236	19.52	1.17 ± 0.356	18.43

***Digestion***					
tyrosine-protein kinase	-1.31 ± 0.000				

***DNA related***					
C14orf124 protein	1.07 ± 0.060				
zinc finger protein	-1.18 ± 0.070				

***Metabolism***					
alcohol dehydrogenase (acceptor)	**2.20 ± 0.005**	1.44 ± 0.285	28.36	**2.08 ± 0.030**	30.64
protease inhibitor 1	1.52 ± 0.010	1.04 ± 0.175	19.56	1.17 ± 0.305	16.42
Serine/threonine-protein kinase polo	-1.20 ± 0.045				
short-chain dehydrogenase	1.15 ± 0.175				
protein disulfide isomerase	1.16 ± 0.020				

***Signaling***					
putative G-protein coupled receptor	-1.64 ± 0.110	1.05 ± 0.245	27.92	-1.41 ± 0.150	30.15

Results are shown for animals grown on bacterial diet, where bacteria-free diet treatments were set to 1 (values are mean ± SD).

### Differential gene expression in *T. ni *eggs laid by parents grown on different diets

Our analysis shows that a number of defense related genes are differentially expressed between eggs laid by parents grown on bacterial (EB) or bacteria-free (EN) diet. Cytochrome C oxidase and GST1 are highly expressed in EB eggs, whereas a BCP inhibitor-like gene and Mn superoxide dismutase transcripts are more abundant in EN eggs [see additional file 2]. GST1, like cytochrome C, belongs to a large family of proteins also involved in various detoxification processes [25]. BCP inhibitor is a cysteine proteinase inhibitor, which has been isolated and characterized from *Bombyx mori *eggs and is known to be involved in degradation of yolk proteins [34]. We also found two developmental gene transcripts highly abundant in EB eggs, namely titin1 which is known to be important in muscle development [35] and a hypothetical chitin binding domain containing protein. In addition to developmental genes we have also identified two genes with DNA binding function to be highly expressed in EN and EB eggs, namely ORF 29 protein and histone H2B protein. As already seen in the larvae, also in the eggs a number of ribosomal proteins showed differential expression depending on the diet [see additional file 2].

Taken together, these results indicate that environmental conditions experienced by larvae of the parental generation can have substantial effects on the physiology of the next generation, and that these effects can be measured at the gene expression level. If expression differences in eggs carry over into the larvae that develop from them, this would support the idea that parents are able to prime their offspring against possible environmental stressors, like increased microbial content, but probably also other factors. The phenomenon of trans-generational priming of immunity has been reported for insects in studies that show increased survivorship or tolerance of infection among offspring of pre-exposed parents; however the underlying genes have not yet been investigated [36-39]. In the present study we show that trans-generational priming of genes expressed in the eggs can be caused by exposure to bacteria in the parental diet. In a separate study we have extended these findings to genes expressed in larvae of the offspring generation. These findings draw attention to the importance of the parental environment, as one of the factors shaping the phenotype of the organism.. It can be one of the major sources for the phenotypic variation of several physiological features influencing fitness and reproduction. Trans-generational effects require more attention in studies of the factors shaping the ecology and physiology of organisms.

## Conclusion

The ecophysiology of any organism is a complex and multifaceted set of processes involving responses to all possible changes in biotic and abiotic environmental factors. Important biotic stress related effectors of the physiology of any organism are diet and defense linked changes in habitat. Innate immunity is a central part of the insects' defense mechanism for dealing with physiological adaptations to biotic stressors in the form of parasites and pathogens. The immune regulatory pathway cascades include a huge number of different cellular and humoral components, many of which are in tight and complex relation to other physiological processes.

Here we report a comparative analysis of differentially induced transcripts for *T. ni *larvae and eggs laid by parents grown on bacterially challenged diet. We selected a differential gene expression study method that does not require previous sequence information, as we use a non-model Lepidopteran species. Comparing transcripts of whole larvae fed on three different diets shows that changes in gene expression are connected with different processes related to metabolism and homeostasis. We also detected several immunity related genes with our differential gene expression analysis. Gloverin, HDD1 and hemolin have been found to be highly abundant also in midgut tissue of bacterial feeding larvae [2]. Changes in immune status are accompanied by alterations in the expression of genes coding for diverse physiological processes. We were able to identify a number of genes showing diet dependent expression, linked to an adjusted physiological status. We observed different expression patterns for a number of classically stress-related genes among others. This is in good correspondence with studies indicating that a variety of stresses can affect immune function in insects [40] and that immune response and alteration of immune status are stressful for an organism [27,28]. It is also clear that these differences are not necessarily the same in different life stages and/or tissues. Furthermore, gene expression in eggs produced by individuals exposed to dietary bacteria was affected in some cases, indicating the potential for transgenerational transmission of an immune response.

## Abbreviations

GST: glutathione S-transferase; SDR: Short-chain dehydrogenase/reductase; FAD: fatty acid dehydrases; ATP: adenosine triphosphate; NAD: nicotinamide adenine dinucleotide; GMC: glucose-methanol-choline oxidoreductase.

## Competing interests

The authors declare that they have no competing interests.

## Authors' contributions

DF designed and carried out the experiments, performed the analyses and wrote the manuscript. HV helped to design the experiment, carry out the data analysis and with writing the manuscript. DGH participated in data analysis and revised the manuscript. All authors read and approved the final manuscript.

## Supplementary Material

Additional file 1 **RT-qPCR primers used in current study**. Primer sequences used to detect differential gene expression in *T. ni*. Click here for file

Additional file 2 **cDNAs from GeneFishing for *Trichoplusia ni*, combined with consecutive banding pattern on the agarose gel**. Expression data, based on agarose gel banding pattern is shown for different life stages (2B – 2 day old larvae on bacterial diet, 2N – 2 day old larvae on bacteria-free diet, 2P – 2 day old larvae on cabbage plants; 7B – 7 day old larvae on bacterial diet, 7N – 7 day old larvae on bacteria-free diet, 7P – 7 day old larvae on cabbage plants; EN – eggs laid by parents grown on bacteria-free diet, EB – eggs laid by parents grown on bacterial diet). Banding pattern is shown in correlation with band visibility on agarose gel (- ... band is absent, + ... band is visible, ++ ... strongly visible band). Click here for file
