# Supplementary material for: Bacterial feeding induces changes in immune-related gene expression and has trans-generational impacts in the cabbage looper (Trichoplusia ni)
Source: Front Zool. 2009 May 7;6:7. doi: 10.1186/1742-9994-6-7 (PMC2685797; doi:10.1186/1742-9994-6-7)
Supplement: Additional file 2 — cDNAs from GeneFishing for Trichoplusia ni, combined with consecutive banding pattern on the agarose gel. Expression data, based on agarose gel banding pattern is shown for different life stages (2B – 2 day old larvae on bacterial diet, 2N – 2 day old larvae on bacteria-free diet, 2P – 2 day old larvae on cabbage plants; 7B – 7 day old larvae on bacterial diet, 7N – 7 day old larvae on bacteria-free diet, 7P – 7 day old larvae on cabbage plants; EN – eggs laid by parents grown on bacteria-free diet, EB – eggs laid by parents grown on bacterial diet). Banding pattern is shown in correlation with band visibility on agarose gel (- ... band is absent, + ... band is visible, ++ ... strongly visible band). [file 1742-9994-6-7-S2.doc]

| **Cluster** | **Acc. No** | **Best BlastX match** | **Banding pattern on agarose gel** | | | | | | | |
| --- | --- | --- | --- | --- | --- | --- | --- | --- | --- | --- |
|  |  |  | **2N** | **2P** | **2B** | **7N** | **7P** | **7B** | **EN** | **EB** |
| ***Defense and recognition*** | GH270329 | ABQ43785: mitochondrial cytochrome c oxidase subunit VIa [Bombyx mori] | - | + | ++ | - | + | ++ | - | - |
|  | GH270411 | ABV68856.1: gloverin [Trichoplusia ni] | - | ++ | ++ | - | ++ | ++ | - | - |
|  | GH270330 | NP_001106738: ubiquinol  cytochrome c reductase  [Bombyx mori] | - | - | ++ | - | + | - | - | - |
|  | GH270331 | NP_001037299: Mn  superoxide dismutase [Bombyx  mori] | + | + | + | + | + | + | ++ | + |
|  | GH270389 | NP_001040131.1: GST omega1 [Bombyx mori] | + | + | + | + | ++ | + | + | + |
|  | GH270405 | ABV68857.1: Hdd1-like  protein [Trichoplusia ni] | + | + | + | + | + | ++ | + | + |
|  | GH270332 | P_001040251: lectin 4 C-type  lectin [Bombyx mori] | + | - | + | + | + | - | - | + |
|  | GH270378 | ACC91897.1: hemolin [Heliothis virescens] | + | - | - | - | + | + | - | - |
|  | GH270333 | AQ75437: cathepsin L-like protease [Helicoverpa armigera] | ++ | + | - | - | - | ++ | + | ++ |
|  | GH270348 | XP_974384: PREDICTED: similar to homologue of Sarcophaga 26,29kDa proteinase [Tribolium castaneum] | - | - | - | - | - | ++ | - | - |
|  | GH270334 | NP_001106742: cytochrome c oxidase polypeptide Vb [Bombyx mori] | - | - | - | - | - | - | + | ++ |
|  | GH270337 | AAS79891: gst1 [Spodoptera litura] | - | - | - | - | - | - | - | ++ |
|  | GH270339 | NP_001037057: BCP inhibitor  [Bombyx mori] | - | - | - | - | - | - | + | - |
| ***Development*** | GH270340 | BAG30780: muscle protein 20 like protein [Papilio xuthus] | - | - | ++ | - | - | ++ | - | - |
|  | GH270341 | XP_001662353: hypothetical protein AaeL_AAEL012245 [Aedes aegypti], conatins chitin binding domain | + | - | + | ++ | ++ | + | - | ++ |
|  | GH270382 | XP_001988215: GH10690 [Drosophila grimshawi], contains chitin binding domain, Perithrophin A | - | - | - | - | - | - | ++ | + |
|  | GH270345 | NP_001108405: titin1  [Bombyx mori] | - | - | - | - | - | - | + | ++ |
| ***Digestion*** | GH270346 | AAA29341: trypsin [Manduca sexta] | - | - | ++ | - | - | ++ | + | + |
|  | GH270365 | BAD22559: lipase [Antheraea yamamai] | + | + | + | + | ++ | + | - | - |
|  | GH270347 | AAV91434: serine protease 3 [Lonomia obliqua] | + | - | + | + | - | + | - | - |
|  | GH270394 | P35045: Trypsin, alkaline A precursor | - | ++ | - | + | ++ | + | - | - |
|  | GH270343 | XP_972363.2: similar to trypsin-like serine protease [Tribolium castaneum] | + | + | - | - | - | + | - | - |
|  | GH270376 | XP_001650916: tyrosine-protein kinase [Aedes aegypti] | - | - | - | + | - | - | - | - |
| ***DNA related*** | GH270368 | NP_001040495: C14orf124 protein [Bombyx mori] | - | - | ++ | - | - | - | - | + |
|  | GH270385 | XP_001952287: PREDICTED: similar to chromosome 19 open reading frame 29 [Acyrthosiphon pisum] | ++ | ++ | ++ | ++ | ++ | ++ | + | - |
|  | GH270400 | XP_001200852: PREDICTED: similar to histone H2B (aa 1-123) [Strongylocentrotus purpuratus] | ++ | - | - | ++ | ++ | - | - | ++ |
| ***Metabolism*** | GH270353 | NP_001091831: enolase [Bombyx mori] | + | + | ++ | + | + | ++ | + | + |
|  | GH270417 | XP_001960088: GF13192 [Drosophila ananassae], similar to short-chain dehydrogenase | + | + | ++ | + | + | + | + | ++ |
|  | GH270358 | XP_552371: AGAP011872-PA [Anopheles gambiae str. PEST], ubiquitin activating enzyme | + | + | ++ | + | + | ++ | ++ | ++ |
|  | GH270375 | EEA92944.1: alcohol dehydrogenase (acceptor) [Pseudovibrio sp. JE062] | + | + | ++ | + | + | + | + | ++ |
|  | GH270397 | NP_001040294: protease inhibitor 1 [Bombyx mori] | - | - | + | - | - | - | - | - |
|  | GH270384 | NP_001037171: protein disulfide isomerase [Bombyx mori] | - | - | + | - | - | + | - | - |
|  | GH270355 | XP_396707: PREDICTED: similar to Serine/threonine-protein kinase polo [Apis mellifera] | - | - | + | ++ | - | - | ++ | + |
|  | GH270409 | NP_001040233: ATP synthase [Bombyx mori] | - | - | + | ++ | - | - | - | - |
|  | GH270415 | XP_001599579: PREDICTED: similar to ENSANGP00000017562 [Nasonia vitripennis], similar to stearoyl-coa desaturase | + | + | - | - | - | + | - | - |
|  | GH270418 | NP_001040310: light-induced protein-like brain protein 44 [Bombyx mori] | ++ | - | - | ++ | - | - | - | + |
|  | GH270388 | NP_001040436: hydroxysteroid dehydrogenase [Bombyx mori] | - | - | - | - | - | ++ | - | - |
|  | GH270407 | XP_564957: Elongase AGAP007264-PA [Anopheles gambiae str. PEST] | - | - | - | - | - | + | + | - |
|  | GH270433 | ABG77272: ubiquitin-53aa extension protein [Pieris rapae] | - | - | - | - | - | - | + | ++ |
|  | GH270423 | NP_001037047: silk proteinase inhibitor [Bombyx mori] | - | - | - | - | - | - | + | - |
|  | GH270386 | AAL60239: takeout [Aedes aegypti] | - | - | - | - | - | - | ++ | + |
| ***Ribosomal protein*** | GH270406 | ABS57435: ribosomal protein S25 [Heliconius melpomene] | ++ | ++ | ++ | ++ | ++ | ++ | + | - |
|  | GH270431 | AAK92157: ribosomal protein L14 [Spodoptera frugiperda] | + | - | ++ | + | + | ++ | - | - |
|  | GH270381 | AAN86048: ribosomal protein S2 [Spodoptera frugiperda] | - | - | ++ | - | - | - | - | - |
|  | GH270374 | AAL26578: ribosomal protein S3 [Spodoptera frugiperda] | + | + | + | + | ++ | + | + | + |
|  | GH270396 | AAV91399: ribosomal protein 27 [Lonomia obliqua] | + | + | + | + | + | + | ++ | - |
|  | GH270410 | NP_001091753: ribosomal protein L36A [Bombyx mori] | ++ | - | - | + | + | - | - | ++ |
|  | GH270377 | NP_001037570: ribosomal protein S15A [Bombyx mori] | - | - | - | - | - | - | ++ | + |
|  | GH270416 | AAV91403: ribosomal protein 5 [Lonomia obliqua] | - | - | - | - | - | - | + | - |
| ***Signaling*** | GH270352 | NP_724186: Paxillin [Drosophila melanogaster] | + | + | ++ | + | + | ++ | - | + |
|  | GH270363 | XP_974400: PREDICTED: similar to lots wife CG33968-PA, Nose resistant to flouoxetine family member [Tribolium castaneum] | + | + | + | + | + | - | - | + |
|  | GH270422 | XP_001599614.1: signal peptidase 12kda [Nasonia vitripennis] | + | + | - | - | ++ | + | + | + |
|  | GH270420 | NP_001040123: electron-transfer-flavoprotein beta polypeptide [Bombyx mori] | - | - | - | - | - | - | - | + |
| ***Unknown*** | GH270366 | XP_001604274.1: similar to CG12009-PA [Nasonia vitripennis] | + | ++ | + | - | - | + | - | - |
|  | GH270399 | XP_001121582: PREDICTED: similar to CG10710-PA [Apis mellifera] | ++ | + | + | ++ | + | + | + | ++ |
| ***No significant match*** | GH270336 | YP_001329156: glyoxalase/bleomycin resistance protein/dioxygenase [Sinorhizobium medicae WSM419] | ++ | + | ++ | - | - | - | ++ | + |
|  | GH270350 | XP_001421381: predicted protein [Ostreococcus lucimarinus CCE9901] | + | + | ++ | + | ++ | ++ | + | + |
|  | GH270359 | XP_001759276: predicted protein [Physcomitrella patens subsp. patens] | + | + | ++ | + | + | + | + | ++ |
|  | GH270390 | XP_001717683: PREDICTED: hypothetical protein [Homo sapiens] | + | + | ++ | + | + | + | ++ | ++ |
|  | GH270429 | NP_001040436: hydroxysteroid dehydrogenase [Bombyx mori] | - | + | ++ | - | + | ++ | - | - |
|  | GH270401 | P_001943585: PREDICTED: similar to zinc finger protein 624 [Acyrthosiphon pisum] | + | - | ++ | - | - | - | - | - |
|  | GH270403 | EAW95139.1: serine arginine repetitive matrix 1 [Homo sapiens] | + | - | ++ | - | - | - | - | - |
|  | GH270428 | YP_001744939: hypothetical protein EcSMS35_2915 [Escherichia coli SMS-3-5] | + | - | ++ | - | - | - | - | - |
|  | GH270387 | NP_927355: hypothetical protein glr4409 [Gloeobacter violaceus PCC 7421] | + | - | ++ | - | - | - | - | - |
|  | GH270403 | ZP_01465929: hypothetical protein STIAU_3143 [Stigmatella aurantiaca DW4/3-1] | + | - | ++ | - | - | - | - | - |
|  | GH270351 | BAB89324: putative G-protein coupled receptor [Homo sapiens] | + | - | ++ | + | + | + | - | - |
|  | GH270356 | XP_001647881: Gustatory receptor 61a, putative [Aedes aegypti] | ++ | + | + | ++ | + | + | + | ++ |
|  | GH270389 | ABU41034: hypothetical protein [Lepeophtheirus salmonis] | + | + | + | + | ++ | + | + | + |
|  | GH270395 | AAA26387: outer membrane protein A [Rickettsia akari] | + | + | + | ++ | ++ | + | - | - |
|  | GH270424 | ZP_02747435: two-component sensor histidine kinase [Clostridium difficile QCD-63q42] | - | + | - | - | - | - | - | - |
|  | GH270342 | XP_001867382: myosin I [Culex pipiens quinquefasciatus] | ++ | + | - | - | + | + | - | - |
|  | GH270362 | YP_001910237: putative secretion/efflux abc transporter, ATP-binding protein [Helicobacter pylori Shi470] | ++ | - | - | + | + | + | - | - |
|  | GH270338 | CAA43583: hydroxyproline-rich glycoprotein [Oryza sativa (indica cultivar-group)] | - | - | - | - | - | - | ++ | + |
|  | GH270335 | XP_001952211: PREDICTED: similar to corneal wound healing-related protein [Acyrthosiphon pisum] | - | - | - | - | - | ++ | - | - |
|  | GH270344 | XP_001017307: Chitinase class I family protein [Tetrahymena thermophila SB210] | - | - | - | - | ++ | - | - | - |
|  | GH270413 | NP_508266: Serpentine Receptor, class H family member (srh-19) [Caenorhabditis elegans] | - | - | - | - | - | - | + | ++ |
|  | GH270391 | XP_318947: Porin AGAP009833-PA [Anopheles gambiae str. PEST] | - | - | - | ++ | ++ | - | - | - |
|  | GH270404 | XP_821286: hypothetical protein Tc00.1047053506401.350 [Trypanosoma cruzi strain CL Brener] | - | - | - | ++ | ++ | - | - | - |
|  | GH270367 | YP_812104: hypothetical protein LACR_2573 [Lactococcus lactis subsp. Cremoris SK11] | - | - | - | - | - | - | - | + |
|  | GH270426 | XP_001652430: hypothetical protein AaeL_AAEL001147 [Aedes aegypti] | - | - | - | - | - | - | + | ++ |
|  | GH270432 | ZP_01222271: hypothetical protein P3TCK_18689 [Photobacterium profundum 3TCK] | - | - | - | - | - | - | + | - |
|  | GH270357 | XP_976032: PREDICTED: hypothetical protein [Tribolium castaneum] | - | - | - | - | ++ | + | ++ | - |
| ***Hit only against EST*** | GH270392 | EB827756: 1151973 KZ03 Plodia interpunctella cDNA clone 584001, mRNA sequence | ++ | + | ++ | ++ | + | ++ | - | - |
|  | GH270360 | Sf1F01923-3-1 Spodoptera frugiperda Fat Body cDNA Library Spodoptera frugiperda cDNA, mRNA sequence | + | + | ++ | + | + | ++ | + | + |
|  | GH270373 | CF258147: 90 Trichoplusia ni fifth instar digestive system cDNA library Trichoplusia ni cDNA, mRNA sequence | + | + | + | - | + | ++ | - | - |
|  | GH270371 | EY265223: BF01036X1D07.f1 Normalized subtracted keck library BF01 Danaus plexippus cDNA clone BF01036X1D07.f1 5, mRNA sequence | - | - | + | - | - | - | + | + |
|  | GH270425 | AT001017: AT001017 Bombyx mandarina library (Hwang JS) Bombyx mandarina cDNA clone H340, mRNA sequence | - | - | + | + | + | + | - | - |
|  | GH270354 | TN-LN-384-G-03-libF_J07 Trichoplusia ni whole larvae normalized cDNA library | - | + | - | - | + | - | + | - |
|  | GH270398 | FF375565: TN-28-lipF_D07 Trichoplusia ni larval non-normalized cDNA library Trichoplusia ni cDNA clone TN-28-lipF_D07 5', mRNA sequence | + | + | - | + | ++ | + | + | + |
|  | GH270412 | FF377777: TN-58-pDNR-lipF_N24 Trichoplusia ni larval non-normalized cDNA library | ++ | - | - | + | + | - | - | ++ |
|  | GH270419 | FF377954:TN-60-pDNR-lipF_N13 Trichoplusia ni larval non-normalized cDNA library Trichoplusia ni cDNA clone TN-60-pDNR-lipF_N13 5', mRNA sequence | - | - | - | + | - | - | - | - |
|  | GH270379 | EX212089: EST # 0000117 Spodoptera litura oxidative-stress responsive cDNA library Spodoptera litura cDNA clone SL-470, mRNA sequence | - | - | - | - | - | - | + | - |
| ***No hit at all*** | GH270393 | No hit against EST databases as well | - | + | + | + | + | + | - | - |
|  | GH270408 | No hit against EST databases as well | ++ | + | + | ++ | + | + | - | ++ |
|  | GH270372 | No hit against EST databases as well | - | - | + | - | - | - | - | - |
|  | GH270427 | No hit against EST databases as well | - | - | + | - | - | - | + | + |
|  | GH270364 | No hit against EST databases as well | + | + | - | + | + | - | ++ | ++ |
|  | GH270421 | No hit against EST databases as well | ++ | - | - | ++ | - | - | - | ++ |
|  | GH270349 | No hit against EST databases as well | ++ | - | - | ++ | - | - | - | ++ |
|  | GH270369 | No hit against EST databases as well | - | - | - | - | - | + | + | - |
|  | GH270370 | No hit against EST databases as well | - | - | - | - | - | - | + | + |
|  | GH270414 | No hit against EST databases as well | - | - | - | - | - | - | + | + |
